# There and Back Again: A Forty-Year Perspective on Physician Nutrition Education

**DOI:** 10.1016/j.advnut.2024.100230

**Published:** 2024-04-24

**Authors:** Jaclyn Lewis Albin, Olivia W Thomas, Farshad Fani Marvasti, Jo Marie Reilly

**Affiliations:** 1 Departments of Internal Medicine and Pediatrics, the University of Texas Southwestern Medical Center, Dallas, TX, United States; 2 Boston Medical Center, Boston, MA, United States; 3 Department of Family, Community, and Preventive Medicine, University of Arizona College of Medicine-Phoenix and School of Nutritional Sciences and Wellness, College of Agricultural, Life and Environmental Sciences, University of Arizona, Tucson, AZ, United States; 4 Clinical Family Medicine and Population and Public Health, Keck School of Medicine of University of Southern California, Los Angeles, CA, United States

**Keywords:** nutrition education, interprofessional, diet-sensitive disease, culinary medicine, undergraduate medical education, graduate medical education

## Abstract

Medical education faces an urgent need for evidence-based physician nutrition education. Since the publication of the 1985 National Academies report “Nutrition Education in the United States Medical Schools,” little has changed. Although several key efforts sought to increase nutrition content in undergraduate medical education over the past 40 y, most medical schools still fail to include the recommended minimum of 25 h of nutrition training. Without foundational concepts of nutrition in undergraduate medical education, graduate medical education unsurprisingly falls short of meeting patient needs for nutritional guidance in clinical practice. Meanwhile, diet-sensitive chronic diseases continue to escalate, although largely preventable and treatable by nutritional therapies and dietary lifestyle changes. Fortunately, recent recognition and adoption of Food is Medicine programs across the country increasingly connect patients with healthy food resources and nutrition education as core to their medical care, and physicians must be equipped to lead these efforts alongside their dietitian colleagues. Filling the gap in nutrition training will require an innovative and interprofessional approach that pairs nutrition with personal wellness, interprofessional practice, and community service learning. The intersectional benefits of connecting these domains will help prepare future physicians to address the social, behavioral, and lifestyle determinants of health in a way that recognizes nourishing food access as a core part of clinical practice. There are numerous strategies to integrate nutrition into education pathways, including didactic and experiential learning. Culinary medicine, an evidence-based field combining the culinary arts with nutritional science and medicine, is 1 promising educational framework with a hands-on, interprofessional approach that emphasizes community engagement. Advancing the critical need for widespread adoption of nutrition education for physicians will require support and engagement across societal stakeholders, including co-leadership from registered dietitian nutritionists, health system and payor reform, and opportunities for clinical innovation that bring this essential field to frontline patient care.

**Statements of significance**

The impact of a nourishing dietary pattern on prevention and treatment of disease is increasingly recognized, leading to rapid growth of multisector food is medicine programs. This movement necessitates urgent attention to the role of medical education in equipping physicians to integrate interprofessional nutritional care, and we review the historical efforts of medical education in nutrition and explore feasible paths to advance the work.

## The Historical Call for Nutrition Training in Medical Education

In 1985, the National Academy Press published an extensive report titled “Nutrition Education in United States Medical Schools,” detailing a need for minimum standards in nutrition education for physicians-in-training [1]. The preface to the 140-page document details the increasing public demand for evidence-based nutrition guidance from medical professionals and summarizes the core goals of the report, which include an assessment of nutrition education strategies already in use, associated successes and failures, and a reported percentage of medical schools teaching nutrition. If written in the present day, the brilliant executive summary of the report would remain just as timely, relevant, and urgent.

The 1985 report’s authors noted increased attention to nutrition at scientific conferences as well as through congressional channels; we once again have both scientific and political emphasis [[2], [3], [4], [5], [6], [7]], including professional conference inclusion of nutrition topics by interprofessional groups (outside of nutrition professionals). The sense of urgency articulated in the report was rooted in public awareness about the intersection of nutrition and health, as well as the many factors driving a need for improved disease prevention strategies. Poignantly, they speak to the need for nutrition education as a long-standing concept victimized by the near resolution of nutritional deficiency syndromes from a public health perspective and the completion of major vitamin identification from a research angle. Instead of expanding the potential for nutritional interventions as a strategy for prevention and treatment across all fields of medicine, the “application of nutrition in clinical practice received less emphasis as the patterns of medical practice shifted from comprehensive care toward specialization and new technology.” Nearly 40 y later, very little has changed in regard to the robust inclusion of nutrition in medical training.

## Nutrition Training in Undergraduate Medical Education

The National Academies report [1] tailored nutrition education recommendations focused on undergraduate medical education (UME). Although concurrent and layered nutrition education in graduate medical education (GME) and continuing education is also essential, medical schools can deliver universally relevant core content in a more standardized manner across the 4 y of UME. Over the past 4 decades, several key efforts have been made to improve the uptake of the nutrition curriculum in United States medical schools.

The NIH funded the Nutrition Academic Award from 1998–2005, and 21 recipients, medical school recipients, developed nutrition education strategies, designed assessment tools, and promoted curricular integration [8,9]. Nutrition Academic Award curricular impact included improved nutritional knowledge, counseling confidence, and personal dietary patterns in learners [[10], [11], [12]]. However, the overall program fell short of achieving longitudinal momentum or widespread adoption of nutritional curricula into medical education [4,13,14].

Since that time, experts have continued to describe the paucity and heterogeneity of nutrition education in medical schools [15]. A series of surveys of United States medical schools accredited by the Licensing Council for Medical Education consistently revealed that the majority of medical schools failed to deliver the recommended minimum of 25 h of nutrition training in the 1985 report from National Academies [16,17]. The most recent survey in 2014 had a 91% response rate and discovered that 71% of surveyed medical schools failed to provide the minimum number of hours, and where curricula did exist, schools self-reported mostly preclinical nutrition courses and little clinical practice application [18]. As medical advances continue to add new knowledge domains, educational real estate struggles to keep up and further diminishes the ease of integrating important content, including the foundational role of nutrition in many disease states.

Given the challenges described, medical educators championing nutrition education have employed creative strategies. For example, medical students viewing a recorded lecture followed by nutritional counseling role play with students with diabetes increased medical student counseling confidence and positive impressions of collaboration with dietitians [19]. At 1 medical school, a brief virtual nutrition education intervention during a clinical clerkship enabled students to apply counseling directly to patient care encounters [20]. Expert educator groups have described other nutrition curricular approaches and lessons learned, calling for improved coordination of educational efforts [21].

Recently, culinary medicine has emerged as a hands-on, practical, and engaging method of teaching nutrition concepts [[22], [23], [24], [25]]. Ripe for educational innovation, the culinary medicine model allows for interprofessional learning, builds community, and promotes the personal wellness of students [[26], [27], [28]]. These factors may be uniquely contributing to the increasingly widespread adoption of culinary medicine as a nutrition education strategy, particularly as it advances to practical applications in patient care [[29], [30], [31], [32], [33], [34], [35]].

### Nutrition training in GME

Ideally, nutrition in GME builds on an established UME foundation and deepens content relevant to the chosen specialty and practice setting. If trainees enter GME programs with the core ability to appraise nutrition literature and translate core principles to nutritional diseases, including chronic disease prevention and management, the ability to impact population health will advance. Most physicians do not need the detailed nutrition foundation of registered dietitian nutritionist (RDN) training but rather a core foundation of evidence-based knowledge, collaboration strategies for interprofessional team-based care, best practices for referral to nutrition professionals, and the ability to deliver practical, timely food advice that applies to their patient population.

Studies assessing the nutrition confidence and skill of primary care, obstetrics, and surgical residents consistently demonstrate a lack of preparation to meet the nutrition needs of patients across a variety of clinical scenarios [[36], [37], [38]]. Fortunately, enhancing nutrition education and other lifestyle-related topics enhances the self-efficacy of trainees to perform counseling [39]. To optimize both the capability and motivation of nutrition interventions in primary care fields, nutrition training optimally begins in UME and continues to build depth in GME [40,41].

Beyond primary care training, several specialties naturally necessitate advanced nutrition knowledge, given its broad relevance to patient populations. For example, cardiologists care for patients with many diet-sensitive diseases, such as lipid disorders, coronary artery disease, and heart failure. However, few cardiologists reported receiving nutrition education during training, with their subspecialty fellowship years the least likely to have integrated nutrition education [42]. As such, calls for enhanced education highlight the potential for cardiologists to serve as key team members in the delivery of nutrition interventions, given the broad impact of diet on cardiovascular health [43].

Similarly, physicians trained in endocrinology care for a complex array of patients with metabolic diseases, most notably type 2 diabetes. However, nearly half of surveyed endocrinology fellowship programs did not offer nutrition education to their trainees, and the same study noted that the programs that did offer nutrition training overly focused on didactic content [44]. The authors emphasized the need for more engaging and interprofessional nutrition education for future endocrinologists [44].

Fellowship training in gastroenterology does not appear to better equip trainees to care for diet-related diseases. An assessment of training for gastrointestinal physicians in the United Kingdom noted the slow progress of nutrition education and the long overdue need to advance this domain in the context of the public expectations for doctors’ nutrition knowledge [45]. Similarly, 95% of gastroenterologists and fellowship program leaders in Canada agree that nutrition education should be standardized in fellowship, but only 36% of surveyed fellowship required this training [46]. Notably, there is sparse literature from the United States on this topic.

Beyond the obvious specialties that routinely care for patients with diet-sensitive diseases, the physician’s ability to support patient adherence to a nourishing dietary pattern also has niche implications, such as in the field of renal transplantation and the subsequent care of donors [47]. In fact, the intersection of nutritional health and surgical outcomes and intensive care is increasingly recognized, deepening the value of nutrition education for surgical fields, as well [[48], [49], [50], [51]]. There are few GME fields that lack the need for a basic foundation of nutrition knowledge layered with specialty-specific tailored content.

## The Current Need for Nutrition Education

The science of nutrition and its varied applications to health is as complex and specialized as it is broad and widely relevant. Although historic nutritional deficiency syndromes have largely resolved in the United States with efforts to fortify common foods, expand dietary variety, and utilize supplements when needed, many Americans do not meet the recommended dietary guidelines across the lifespan, as reflected by poor scores on the Healthy Eating Index [52]. Improving the quality of dietary patterns across the population includes addressing food and nutrition security and the complex reasons why access to nourishing food is not equitable in the United States [53,54].

The suboptimal diets of most Americans can impact an individual’s overall quality of life and lead to substantial social and economic consequences on a national scale. Diet-related diseases, including cardiovascular diseases, diabetes, and cancer, are the leading causes of illness, disability, and death in the United States and significantly contribute to the $4.1 trillion spent annually on healthcare costs [55]. The incidence of these noncommunicable diseases is on the ascent, and the prevalence of ≥1 chronic disease in individuals aged ≥50 y is projected to surge by 99.5%, increasing from 71.5 million in 2020 to a projected 142.7 million by 2050 [56].

Poor dietary patterns and the associated risk of chronic disease are related to social, economic, and geographic factors that disproportionately affect marginalized communities [57]. Social determinants of health, or the conditions in which people live, learn, and work, can lead to unmet health-related social needs, such as access to adequate and preferred nutritious foods. Considering the structural drivers of health, addressing nutrition-related health disparities requires a comprehensive approach that extends beyond individual choices to encompass broader societal and environmental considerations.

To address this complex issue, health systems and payers have shifted over the last decade toward value-based approaches and the establishment of accountable care organizations, requiring healthcare providers to enhance patient outcomes while minimizing costs through preventative and lifestyle interventions that address social needs [58,59]. More recently, the Centers for Medicare and Medicaid Services and the Joint Commission have mandated hospital quality measures linked to social drivers of health and health equity standards, leading to the mass adoption of social needs screening and investment into associated resources and care management [[60], [61], [62]]. Although improved screening is a vital foundation, sustainable responses to social needs, such as food insecurity, require an educational foundation in nutrition for leaders to innovate and guide these interventions.

To further address health inequities, the Biden administration convened the White House Conference on Hunger, Nutrition, and Health in September of 2022, with stakeholders including elected officials, advocates and activists, and leaders in business, faith, and philanthropy who mobilized to discuss strategies to end hunger, improve health, and prevent chronic disease risk for Americans by 2030 [7]. The conference catalyzed a national movement called Food is Medicine (FIM), or sometimes Food as Medicine, that intersects healthcare with food and nutrition resources and suppliers to address the need for access to medically tailored foods, meals, and education and skill-building interventions. The FIM movement is gaining momentum due to the emerging research that supports improved patient outcomes and the prevention of future hospitalizations or worsening health conditions [54,63]. For example, the national implementation of the produce prescription programs for people with diabetes and food insecurity could result in 292,000 averted cardiovascular events [64], and prescribing mobility-limited patients with diet-sensitive conditions medically tailored meals could result in ∼1.6 million averted hospitalizations [65]. These drastic improvements to healthcare utilization could reap profound economic benefits and savings in healthcare spending in addition to a clear reduction in morbidity.

Changes to the American health system have directly impacted the practice of medicine and the educational approaches to train future clinicians. In 2022, the United States House of Representatives passed House Resolution 1118, which calls on medical schools, GME programs, and other health professional training programs to provide meaningful nutrition education [66]. In March 2023, the resolution was followed by a Summit on Medical Education in Nutrition hosted by the Accreditation Council for GME (ACGME), the Association of American Medical Colleges, and the American Association of Colleges of Osteopathic Medicine; the Summit focused on strategies to advance nutrition education across the education continuum [2].

In response to this rapidly evolving context, many medical schools are adopting innovative approaches to empower aspiring physicians with the skills and knowledge necessary for delivering comprehensive and holistic patient care, encompassing nutrition and lifestyle interventions. One medical school addressed curricular space constraints by weaving a nutrition “thread” through the entire curriculum rather than developing a standalone nutrition course [67]. Given the ease of access and inherent flexibility, the use of online curricular resources has become increasingly relevant and offers an opportunity for low-resource nutrition education. An online module followed by a nutrition knowledge assessment and Objective Structured Clinical Evaluation to assess nutrition counseling skills proved feasible and well-received at 1 medical school [68]. Another study assessed a 3-h nutrition module and demonstrated sustained nutrition knowledge and positive attitudes about nutrition in practice 3 mo after the online course completion [69]. As previously described, many medical schools now use culinary medicine to engage students through experiential learning and provide tangible skills around food and nutrition [23,24], a vital strategy to ensure learners are equipped to design and prescribe evidence-based FIM programs. Recognizing the historic inadequacy of nutrition training, innovative culinary medicine programs also influence clinicians in practice [70]. Additionally, culinary medicine encourages interprofessional collaborations and elevates the role of nutrition and culinary experts and RDNs [71,72].

## Notable Challenges, Barriers, and Opportunities

### Challenges and barriers

Moving the needle forward on physician nutrition education training has been hampered by the lack of oversight, guidance, and mandates by the bodies that oversee and accredit their training [5,73,74]. Specifically, there are limited or absent current requirements by the Liaison Committee on Medical Education (LCME) or ACGME for specific learner training on lifestyle management, exercise, and nutrition, leading to heterogenous elective-based experiences that vary widely across programs [[75], [76], [77]]. With an often packed medical school and residency curriculum and no required curricular focus on nutrition, there is no “stick” to reinforce the “carrot” of the call for a nutrition curriculum recommended by the 1985 report [1]. Further, without individual medical school or residency faculty champions, medical schools and residency programs lack the resources and training to support sustainable integration of the nutrition curriculum. For schools and programs that do have faculty champions, the nutrition curriculum is rarely delivered consistently or uniformly across programs due to a lack of support for logistical and training needs, and clinical mentors, in many cases, lack sufficient base-level nutrition education to effectively train students.

Physician education is highly incentivized and regulated throughout training. This begins with medical school examinations, national licensing examinations, and postresidency specialty board and fellowship examinations. It continues with specialty board recertification examinations and continuing medical education requirements tied to specialty board maintenance. Historically, nutrition, exercise, and lifestyle medicine have not been emphasized in licensing examinations, except those tied to vitamins and usually with a disease-specific state [4,22,78]. Additionally, many medical schools no longer allow faculty to write their own examination questions, relying on national standardized board examination topics and questions that neglect nutrition. This lack of testing devalues nutrition education and continues to be a barrier to standardized nutrition education for early physician trainees.

Lack of testing further contributes to the de-incentivization of nutrition learning, a problem also reinforced by postgraduate board examinations, particularly the case in specialty fields such as gastroenterology, cardiology, and endocrinology [[42], [43], [44],^46^,^79^,^80^]. As previously described, these fields have a preponderance of patients with secondary and tertiary diet-sensitive health problems, including diabetes, heart disease, hypertension, inflammatory bowel diseases, colon cancer, and fatty liver disease. However, these internal medicine training programs and subspecialty examinations rely more on technical skill acquisition and pharmaceutical management while neglecting emphasis on primary prevention, including training in lifestyle behavioral change, nutrition, chronic disease management, and motivational interviewing. Similarly, vascular surgery training emphasizes the technical skills of amputation and revascularization without lifestyle modification counseling to manage the chronic conditions that contribute to the conditions needing advanced procedures. This reinforces the medical management model of United States health care delivery rather than equipping trainees to lead with an urgently needed lifestyle and health prevention model [[81], [82], [83]].

To address serious public health concerns and crises, multiple states have tied specific supplemental health training to physician licensure and license renewal [84]. Two recent examples tied training for physicians to license renewal requirements include the opioid crisis, mandating opioid and pain management training, and end-of-life and palliative care training. Requiring training often results in the development of multiple course offerings, including free educational resources, and it typically leads to a measurable increase in physician completion of training [85]. Presumably, the education contributes to additional knowledge and competency in the respective required subjects. Notably, training mandates associated with licensure come with a timeline, reinforcing the urgency of addressing physician educational deficiencies and spurring the creation of accessible resources to support the requirements. There has not yet been a similar call for nutrition and lifestyle medicine training mandates despite the vast and well-documented increases in diet-sensitive conditions.

In addition to the described challenges, several additional barriers prevent the implementation and facilitation of nutrition education and impede the effectiveness of preventative care. Fundamental to holistic patient care is a strong primary care base, but the United States health care system has a subspecialty emphasis. Primary care prevention, lifestyle medicine (including nutrition and exercise), community engagement, social determinants of health, and health literacy promotion are all foundational to supporting and preventing the onset and progression of chronic nutritional diseases. There has been a historical lack of support and value for primary care in the United States health care system, limiting prevention-focused physician careers beginning in medical school, and this problem will likely progress rapidly in the context of national physician workforce shortages [86]. Lack of reimbursement for registered dietitian services is also a commonly cited barrier to optimal interprofessional practice, in addition to the inconsistent availability of dietitians in primary care and many subspecialty settings. Without primary care payment reforms and value alignment, including physician compensation for delivering vital health education to patients, such as nutrition-focused care, patient access to these vital services may continue to decline.

A final barrier worth emphasizing is the inadequate focus on collaborative care training in medical education, an omission that has slowed physician trainee preparedness to work in collaborative care teams. Interprofessional health training in nutrition provides an opportunity and supports integrative and holistic patient care, preparing physicians to work with RDNs, diabetic educators, advanced care practitioners, mental health professionals, social workers, and others who work alongside physicians to advance patients’ nutrition self-efficacy and comprehensive health care needs.

### Opportunities

Despite the challenges and barriers to increased nutrition education, there have been some recent changes across the education and policy sectors that invoke hopefulness, including increased national interest in the policy impacts of nutrition training and expansion of governmental and nongovernmental efforts [7,66]. There are a growing number of nutrition curricular resources and support for medical educators to provide nutrition training [[22], [23], [24], [25], [26],^77^,^87^,^88^], increase emphasis on interprofessional practice [72,89], and deliver holistic lifestyle medicine education, residency, and certification opportunities [90,91]. Continued efforts to standardize and advance nutrition competencies across undergraduate, graduate, and continuing education will be vital for sustainable, nimble curricular strategies and the advancement of assessment strategies that truly measure educational outcomes [4,92,93].

## Thematic Successes and Strategies

As medical education real estate seemingly shrinks with more to cover in less time, multimodal strategies that cross-topic domains are essential. In the case of nutrition education, the opportunities to pair core content with interprofessional practice, personal wellness, and service learning are abundant.

Interprofessional practice in nutrition is essential. From an educational perspective, physician learners benefit from early exposure to colleagues in other health professions, and hands-on nutrition education activities deliver an ideal setting for engaging collaboration. One study demonstrated the feasibility of both in-person and virtual culinary medicine courses combining a broad array of students from programs including social work, medicine, nutrition, law, dentistry, and pharmacy [94]. The impact of the interprofessional practice of nutrition extends beyond training environments to patient care and community implementation, inclusive also of food service professionals who ensure that nourishing food tastes delicious [71].

The personal wellness of physicians has been an increasing concern, and strategies to advance the wellness of students and trainees remain key priorities [[95], [96], [97]]. Nutrition education uniquely offers an opportunity to advance personal dietary wellness while building skills in patient care counseling. One study found that students participating in a culinary medicine elective increased confidence in patient nutrition counseling as well as their own cooking skills; they also increased fruit and vegetable intake [98]. Other educators have demonstrated similar results, showing the intersectional benefits of nutrition education on counseling confidence, personal dietary patterns, and interprofessional training [99].

Pairing nutrition education with service learning has the potential to meet workforce needs that advance community health while providing practical, hands-on nutrition training for health professional learners. In 1 study, educators found that medical students leading community-based nutrition interventions reported sustained nutrition knowledge and confidence above baseline, including sustained changes to their personal dietary patterns [100]. Similarly, another recent study showed that a medical student elective in culinary medicine increased self-reported confidence in discussing nutrition with patients and its application to health, and students subsequently had the opportunity to apply their knowledge in community settings that included middle school students [29]. The next frontier of this work includes developing best practices for equipping and then deploying medical students and postgraduate trainees to deliver community-based nutrition interventions.

In spite of many successes, culinary medicine integration poses key limitations given the need for significant resources, including space, equipment, food, and instructor time. Pairing other nutrition education strategies with hands-on experiences can offer a variety of approaches based on resources and needs. Additional research across all nutrition education strategies is needed to define educational standards and optimal assessment methods.

## Future Directions and Recommendations

Despite well-thought-out efforts to enhance nutritional content in medical education since the publication of “Nutrition Education in United States Medical Schools” in 1985 [1], there continues to be a paucity of foundational nutritional content in UME and clinically relevant GME nutritional education. With the persistent “slow” pandemic of chronic disease linked to poor dietary intake and preventable, premature disease and death, the urgency for nutrition in medical education cannot be overstated. This crisis presents an opportunity for thoughtful innovation and change, emphasizing key curricular areas that can make a lasting impact.

First, based on emerging data from numerous curricular innovations, a multimodal, longitudinal, interprofessional approach to medical nutrition education is both sustainable and comprehensive. For UME, this strategy begins with basic science nutritional content in preclinical years and develops further with clinical cases and hands-on culinary skills during clerkships, consolidating knowledge in an incremental way. Including additional chronic disease management and motivational interviewing skills in postgraduate and fellowship training for primary care and key internal medicine and surgical subspecialties is critical. Nutrition counseling skills must be valued and incentivized in addition to technical skill training, a goal likely advanced by the growth of FIM strategies in clinical care, necessitating ongoing study of sustainable approaches. Further, these nutrition strategies must be aligned with the accrediting bodies that dictate undergraduate and GME standards.

Second, creating educational approaches with an interprofessional framework will not only address curricular deficits in nutrition but will also offer a content area that is ripe for modeling interprofessional team-based care. This training includes core collaborations with RDNs, speech pathologists, physical and occupational therapists, social workers, pharmacists, behavior change experts, and other colleagues. Dietitian colleagues can and should take a key leadership role in supporting physician nutrition education due to their subject matter expertise. Physician-dietitian teams can build successful collaborations, offering the best opportunity for enhanced patient referrals and access to nutrition care.

Third, nutrition education content must include more than basic biochemistry and discussion of nutrients. Although not all medical students need extensive nutrition training, integrating nutrition principles into UME through GME remains crucial for best practices in clinical care and addressing the growing chronic disease epidemic. Current efforts are underway to organize core topics for nutrition competencies in response to the previously described ACGME Nutrition Summit [2]. The use of applied learning techniques, such as culinary medicine, appears particularly effective and well-received among learners. Nuanced skills and expertise can be further developed in later stages of training, especially at the GME level. Further, it must be incentivized through national medical student board examinations as well as through postresidency specialty, fellowship, and recertification examination reform. Learners need training to advance the direct clinical application of nutrition in the context of chronic diseases, including cancer, obesity, diabetes, hypertension, and hyperlipidemia. Utilizing multiple novel learning strategies like culinary medicine and service learning enhances didactic nutrition education by providing a hands-on approach. This strategy allows learners to develop key skills directly applicable to patient care and lead evidence-based discussions about nutritional science and dietary strategies for health. Culinary medicine also enables learners to amplify their own personal wellness through dietary improvement and peer interaction, another area of opportunity for medical education. To prevent physician burnout, trainees must learn self-care strategies and build nutrition knowledge. Learning healthy food preparation skills supports student and resident health and informs authentic patient encounters.

Fourth, the relevance of food policy in determining equitable access to healthy food and the health impact of food insecurity must accompany nutritional science content. In this way, nutrition can be understood in the context of other social, behavioral, and lifestyle determinants of health. Embedding learners in community settings allows learners to see nutrition in this broader context. Service-learning functions as an important way to empower learners to make an impact in the community while training in the real world.

Fifth, primary care payment reforms and value alignment, including physician compensation for delivering vital health education, are critical to holistic patient care. These opportunities include clinical nutrition innovation and dietary strategies for health within existing payor models, such as the interprofessional Culinary Medicine eConsult [101]. Prior studies describe group medical care to build patient self-efficacy and behavior change in a feasible and creative context [[102], [103], [104], [105]]. Efforts should also include the standard integration of referrals to and reimbursement for RDNs to further support and enhance the nutrition conversations with referring clinicians. Advancement of these approaches will likely lead to improved patient satisfaction and engagement, value [59], and quality of care delivery.

Lastly, although these recommendations present an exciting prospect in medical education, success will require a conscious allocation of financial resources and realignment of incentives to ensure enduring impact and sustainability. This includes public and private sector investment. The recent United States House Resolution on nutrition education in medical training included mention of Medicare’s substantial support for GME. Each institution must assess the opportunities for innovation and commitment to this essential transformation within medical education. As physicians, we fundamentally seek to improve the lifespan and health span [106] of our patients. This cannot be achieved without long-overdue attention to the profound role of dietary quality on the health of our patients, friends, neighbors, family members, and ourselves.

## Author contributions

The authors’ responsibilities were as follows – JLA: was invited to author a manuscript on the history, progress, and need of physician nutrition education; JLA: conceptualized the overall design and authored an initial, partial draft; OWT, FFM, JMR: made meaningful contributions to the design and content planning, and all authors wrote various sections of the manuscript. All authors meet the criteria for authorship based on ICMJE, contributed to the critical revision of the overall manuscript, and all authors: read and approved the final manuscript.

## Conflict of interest

The authors report no conflicts of interest.

## Funding

The authors reported no funding received for this study.
